# The Effect of Life Skills Training on the Self-Management of Patients with Multiple Sclerosis

**DOI:** 10.25122/jml-2018-0044

**Published:** 2018

**Authors:** Behzad Hemmatpoor, ALI Gholami, Shima Parnian, Mahnaz Seyedoshohadaee

**Affiliations:** 1. Clinical Research Development Center, Kermanshah University of Medical Sciences, Kermanshah, Iran; 2. Department of Medical-Surgical Nursing, Iran University of Medical Sciences, Tehran, Iran

**Keywords:** multiple sclerosis, psychological training, self-management

## Abstract

**Background:** Training sessions are the most common framework used to provide self-management for patients with multiple sclerosis

**Objective:** This study was conducted to determine the effect of life skills training on the self-management of patients with multiple sclerosis. Findings showed that life skills training had a significant effect on the overall self-management score; thus, self-management of patients increased after training life skills (F = 25.1821, P <0.01).

**Materials and Methods:** This semi-experimental study was conducted on 80 subjects with MS in the MS Society in Tehran (Iran) in 2016. The experimental group received four one-hour sessions of life skills training. The control group received routine care. Patients of both groups filled MS self-management (MSSM) scales at baseline and a month after the last training session. Independent t-test was used to compare findings between the two groups; pairwise t-test was used to compare results before and after the test. Covariance test was used to present the research results and data was analyzed by using SPSS21.

**Findings:** Mean and standard deviation of age were 32.22±8.88 and 33.02±10.34 in the control and experimental groups, respectively.

**Results:** Comparison of total self-management showed a significant difference between the control and experimental groups after receiving life skill training (P<0.01). Moreover, there was a significant difference in the mean of total self-management in the experimental group before and after the intervention; self-management increased after intervention (p-value<0.001).

**Conclusion:** This study showed the positive effect of life skills training because its main goal is to prepare and help patients solve problems and deal with difficulties resulting from the disease. Therefore, life skill training is suggested for patients suffering from MS.

## Introduction

Multiple sclerosis (MS) is an autoimmune disorder which can be associated with a variety of motor, cognitive and psychiatric symptoms as well as neurological disorders [1]. According to the National MS Society, 400,000 people in the USA and about 2 million people worldwide suffer from MS [2, 3]. In 2010, 5.3 to 74.28 per 100,000 people in different areas suffered from MS in Iran [4]. Most people with MS progressively become disabled over the course of the disease. These disabilities affect the independence of patients [5]. About 60% of people with MS will depend on mobility aids after 20 years on average from the debut of the illness [6, 7]. Self-management is an effective factor in the quality of life of people with MS [8, 1].

Despite the availability of disease-modifying treatment since 1993, MS management has remained a challenge [9]. Although drugs prescribed to treat MS are slightly effective, they have adverse effects and difficult tolerability [10]. Self-management signifies successful management in a disabling condition; it requires special skills, the learning of which helps individuals adapt to, and overcome, specific situations [11]; as a continuous process, it is complementary to medical and clinical management [12] and self-management is a potential approach to reducing symptoms associated with MS [5]. Learning self-management skills applied in everyday life can facilitate adaptation to the disease and reduce the risk of secondary complications. There are few findings which show those interventional strategies effective in enabling patients with MS to learn and use self-management skills for daily life and support [11]. Training and training information is a core element of all self-management interventions. Training sessions are the most common framework used to provide self-management; these often include information about lifestyle, adaptation, and preservation of those styles through psychological strategies [12].

Studies have shown that psychological interventions can improve the physical and psychological well-being of patients with MS by treating mood disorders such as anxiety and depression, improving self-management, promoting self-efficacy and self-esteem, reducing stress, improving consistency skills and general quality of life [13]. A life skills training is a psychological intervention. Life skill is the capability for adaptation and positive behavior which enables one to deal effectively with the needs and challenges of everyday life [14]. This study tends to determine whether life skills training can be useful in improving self-management in patients with MS.

## Materials and Methods

### Sampling Method

The present semi-experimental study (based on intervention, namely group training and having a control group and random allocation of samples), was conducted in order to assess the effect of life skills training on self-management in MS patients admitted to the Tehran Multiple Sclerosis Association in 2016. The researcher selected 89 samples through the available sampling method according to the inclusion criteria; 9 of them were dropped during the research process, and the study was conducted on 80 subjects. Patients included in the association went to the MS community to participate in training classes from Saturday to Thursday, so that the classroom participants were different from day to day. The researcher attended the MS community classes on Sunday and Tuesday for selecting the control group and on Saturday, Monday and Wednesday to select the test group. According to the choice of the test and control group on separate days in order to prevent information bias, it was not possible to allocate the samples randomly. In this way, 46 patients were selected as the test group and 43 subjects were selected as the control group. Among the test group subjects, one individual was removed due to not attending a training session, one of the participants refused to continue the study, and four others left the study due to recurrence of illness and hospitalization. In the control group, there were also 3 cases excluded from the study, all of whom were not included in the second stage due to the recurrence of illness and admission to the hospital.

### Inclusion and Exclusion Criteria

Inclusion criteria included: definitive MS diagnosis by a neurologist; literacy; ability to attend training sessions; absence of disease recurrence over the last three months; absence of pregnancy; no changes in dose of disease-modifying drugs, if taken; absence of other disabling diseases and cognitive involvement; absence of chronic heart and lung diseases; absence of psychiatric disorders and psychiatric drugs referring to self-report and patient records.

Exclusion criteria included: Hospitalization due to recurrence of illness during the study; absence in a session or unwillingness; MS type change to progressive disease during the study; excessive fatigue not allowing the patient to receive training.

### Demographic Questionnaire

The demographic questionnaire contained information on gender, age, education, economic status, marital status, employment, and personal health assessment, history of psychiatric disorders and drug consumption and questions about the MS disease including the type of MS (progressive/relapsing, remitting), the course of the disease and deterioration.

### Multiple Sclerosis Self-Management Scale

The Multiple Sclerosis Self-Management (MSSM) scale was used to evaluate the self-management of patients. MSSM is a multidimensional instrument used to measure self-management knowledge, and behavior among people with MS. This scale was initially developed by Bishop and Frain in 2007. It contains 39 items and 7 factors including treatment adherence, healthcare provider-patient relationship, emotional health and social support, health and symptom awareness, MS knowledge and information, health maintenance behavior, and communication about symptoms/changes. This instrument is scored on a 5-point Likert scale ranging from 1 (strongly disagree) to 5 (strongly agree). Higher scores show better self-management. “Healthcare provider-patient relationship” includes four items (12–19). Treatment adherence includes seven questions (1, 7–10, 15). “Emotional health and social support” includes eight questions (22–26, 28, 30, 35). “Communication about symptoms/changes” includes four questions (6, 11, 13, 14). “Health and symptom awareness” includes five questions [2, 36, 39, 34, 37]. “MS knowledge and information” includes 5 questions (1, 3, 4, 16, 27). “Health maintenance behavior” includes five questions (29, 38, 32, 5, 33) [15].

### Validity and Reliability

Content analysis was used to determine the validity and reliability of the demographic questionnaire as well as the training manual. Bishop and Frain (2007) reported high and acceptable reliability for MSSM (α=.86) [15]. Bishop et al. (2008) used MSSM to measure self-management, perceived control, and subjective quality of life of patients with MS [15]. This scale has not been used in Iran yet. Therefore, validity and reliability were performed in the present study. Initially, the MSSM tool was translated, for which the tool was initially translated by 3 translators (2 translators specializing in medical concepts and a bilingual expert translator), and then translated into Persian; then, they agreed on concepts and the content of the translation in a single session. The text translated by two other bilingual experts was translated into Persian. These translators were unaware of the original questionnaire. Then, in a meeting with translators and researchers, the texts translated into English and Persian were matched to the questionnaire’s original form and agreed on its concepts and terms. Content validity index was used to assess the validity of the questionnaire and the questionnaires were given to 10 faculty members to give their corrective comments after completion; Cronbach’s alpha of the questionnaire, the final rate of which turned out to be a confidence coefficient higher than 0.85%, was evaluated by a specialized professor to determine the reliability of the questionnaire.

### Intervention

The demographic questionnaire and MSSM were given to subjects for a basic assessment of the patients before the training. MSSM was filled again by both control and experimental subjects in the eighth week (because, according to scientific sources, evaluation should be conducted one month after the completion of the training to evaluate the result of the training accurately). Four 1-hour training sessions were administered to the experimental patients (one session per week). At the same time, the experimental group received a training manual and a CD. The manual was based on available information as follows:

1. First session: Multiple sclerosis, its nature, the concept of life skills training and its applications, self-awareness skills
2. Second session: empathy, interpersonal relationships, and effective communication
3. Third session: a reminder of past content; stress coping skills, emotional management, and problem-solving
4. Fourth session: review, decision-making skills, creative thinking, and critical thinking.

All sessions were held while the author and samples were present one hour before the classes (content of the training classes was different and there was no overlapping with the content taught during the research). Moreover, a psychiatric nurse supervised proper administration of sessions. The training was provided by the researcher through lecture, discussion and question and answer. Audiovisual devices, including a video projector, were also used. In addition, the test book was delivered to the test group at the same time. If a subject was absent in a session, he would be excluded from the study. The control group received usual care and filled the scales. The training booklet and the relevant CD were provided for the control group at the end of the study to observe ethics in the research after completing the questionnaires. Then, the filled questionnaires were analyzed.

### Statistical Analysis

Descriptive statistics and the covariance test were used to describe data and reach the research objectives, respectively. Chi-square test, Independent t and paired t-test, pairwise t-test, and Fisher’s exact test were used to achieve objectives and test hypotheses. All data were analyzed by using SPSS, version 21.

### Ethics

This study was approved by the ethics committee (No. IR.IUMS.REC.1394.9311686009) and the research council of the MS Society of Iran. Verbal and written informed consent was taken from the subjects. For morality issues, the training manual and its CDs were given to controls at the end of each session.

## Results

Most respondents in both experimental and control groups were male (55% and 57.5%). Mean and standard deviation of age was 32.22±8.88 in control and 33.02±10.34 in experimental subjects. Chi-square, independent t-test and Fisher’s exact test showed that the two groups did not differ significantly in terms of demographic characteristics (Table 1).

**Table 1:** Comparisons of demographic variables in experimental and control groups: Results of the chi-square test, independent t-test, and Fisher’s exact test (n=80).

Variable		Control group	Experimental group	
Gender (%) n	Male	(57.5) 23	(55) 22	Chi-square test; P-value=0.822; X^2^=0.051; df=1
	Female	(42.5) 17	(45) 18)	
Age (Mean±SD)		32.22 (8.88)	32.02 (10.34)	Independent t test; t=0.371; df=78; p-value=0.712
Education n (%)	Below Diploma	2 (5)	0(0)	Fisher’s exact test: p-value=0.38
	Diploma	25 (62.5)	26(65)	
	Above diploma	13 (25.5)	14(35)	
Health condition n %	Bad	4(10)	3(7/5)	Fisher’s exact test: p-value=0.65
	Good	32(80)	35(87/5)	
	Very Good	4(10)	2(5)	
Disease type n %	Relapsing-remitting	19(47/5)	17(42/5)	Chi-square test; P-value=0.79; X^2^=0.47; df=2
	Primary progressive	15(37/5)	18(45)	
	Secondary progressive	6(15)	5(12/5)	
Time elapsed since the last attack (in months) (Mean±SD)		7.80 (5.91)	7.30 (5.51)	Independent t test; t=0.391; df=78; p-value=0.697
Frequency of disease attacks (Mean±SD)		5.90 (3.40)	5.90 (3.01)	Independent t test; t=0.001; df=78; p-value=0.99

**Table 2:** Mean self-management scores and its dimensions in the control and test groups (after training) -the covariance test

Dimensions	Group	Mean	Std. Deviation
The relationship between the patient and the caregiver	Test	9.9750	3.32425
	Control	8.2000	3.37563
Adherence to treatment	Test	14.9250	6.10333
	Control	12.5750	4.63480
social support	Test	20.6000	7.09207
	Control	16.7500	5.48074
Communicate about symptoms	Test	7.5500	3.36612
	Control	6.9750	3.89929
Awareness of symptoms and health	Test	11.7500	3.06134
	Control	9.5000	1.88108
Knowledge and information about the disease	Test	10.4750	2.80098
	Control	7.2000	2.04061
Healthy Behaviors	Test	16.1500	3.44592
	Control	15.2000	2.70043
Self-management (total)	Test	90.1750	20.22044
	Control	76.4000	15.07384

According to the results of covariance (Table 3), it was concluded that training life skills can affect various dimensions of patient relationships with the caregiver (F = 4/0713, P <0.05), adherence to treatment (F = 0.73323, 01 (P = 0.01), knowledge of the symptoms (F = 17.1961, P <0.01), knowledge and information about the disease (F = 1.09 750, P = 0.01) <), and also the overall self-management score (P = 0.011; P = 0.01), and it can be said that life skills training has increased the scores of these dimensions of self-management.

**Table 3:** The results of the univariate co-variance analysis (ANCOVA)

Dimensions	Source	Type III Sum of Squares	df	Mean Square	F	Sig.
The relationship between the patient and the caregiver	pre-test	443.130	1	443.130	78.939	.000
	Group	26.459	1	26.459	4.713	**.033**
	Error	432.245	77	5.614		
Adherence to treatment	pre-test	295.040	1	295.040	11.385	.001
	Group	189.774	1	189.774	7.323	**.008**
	Error	1995.510	77	25.916		
Social support	pre-test	538.789	1	538.789	15.991	.000
	Group	422.125	1	422.125	12.529	**.001**
	Error	2594.311	77	33.692		
Communicate about symptoms	pre-test	142.570	1	142.570	12.303	.001
	group	1.767	1	1.767	.152	.697
	Error	892.305	77	11.588		
Knowledge and information about the disease	pre-test	18.884	1	18.884	3.001	.087
	group	113.043	1	113.043	17.961	**.000**
	Error	484.616	77	6.294		
Awareness of symptoms and health	pre-test	9.521	1	9.521	1.598	.210
	group	219.000	1	219.000	36.750	**.000**
	Error	458.854	77	5.959		
Healthy Behaviors	pre-test	49.281	1	49.281	5.435	.022
	group	29.580	1	29.580	3.262	.075
	Error	698.219	77	9.068		
Self-management (total)	pre-test	9906.530	1	9906.530	51.192	.000
	Group	4996.834	1	4996.834	25.821	**.000**
	Error	14900.845	77	193.517		

The results showed that life skills training did not affect two dimensions of communication about symptoms (P = 0.055, P> 0.05) and healthy behaviors (F = 3/262, P> 0.05).

## Discussion

The results of the covariance test showed that training life skills has been effective on patient self-management in various dimensions of adherence to treatment, patient relationships with the care provider, social support, knowledge of symptoms and knowledge and information about the disease, but there were no significant impacts on the two aspects of communicating about symptoms of illness and healthy behaviors. Consistent with the above findings, the results of Halloy et al.’s, study showed that psychological counseling reduces the level of non-compliance with treatment for patients with tuberculosis. The main similarity between this research and the present study is the type and content of the intervention, both of which are psychological training. In addition, the two studies have similar inclusion criteria. However, since there has not been a study on the effect of psychological training on adherence to treatment in MS patients, the findings of the present study are groundbreaking, which can be considered a strength. The results of another study by Jangen et al. showed that the primary outcome of self-management turned out to be controlling autonomy and participation, anxiety, depression and cost-effectiveness; a decrease in the pressure of care and increased social support for friends was another favorable finding. The results of Jangen et al.’s research on social support are in line with the findings of the present research, which can be a reason for the emphasis of both studies on the necessity of psychological education. In addition, the criteria for entering the two studies are somewhat similar. The results of a study by Clondry et al. showed that cognitive-behavioral interventions increase the quality of life and psychological well-being of MS patients by reducing negative emotions and promoting mental health and optimism, points which are, in general, consistent with the findings of the present study.

The results of another study by Maguire et al. in Portugal are inconsistent with the findings of the present research. This study showed that, in the field of depression, anxiety and general mental health, perceived stress, and progressive pain were observed, but there was no significant difference between groups of experiment and control concerning social support, cognitive problems and fatigue. Perhaps the reason for this difference is, firstly, that although both programs emphasize the psychological dimension, the educational content of the two programs varies significantly so that the study emphasizes the increasing awareness of various social, intellectual, emotional, and psychological factors. However, the main content of the research is the teaching of 10 life skills. Secondly, the tools used in the other study assess social support, while social support in this study has been measured as one of the seven dimensions of the self-management questionnaire for MS patients [2, 16–18].

The results were significant regarding awareness of symptoms, as well as knowledge and information about the disease. In this regard, due to the trained content that has been targeted in the nature of the disease, such a finding is not expected. According to a search in the databases, the effect of psychological training on awareness of the symptoms and knowledge of MS patients and even other chronic illnesses has not been done, a fact which can be considered another strength of the present study, and growth in both dimensions of self-knowledge and self-awareness has confirmed the effectiveness of self-management skills training. However, this factor did not turn out to be effective on the dimension of health behaviors, which may be due to the inadequacy of training in this dimension or the presence of other interfering factors beyond the control of the researcher. Regarding the fact that there is no study on the effect of psychological training on health behaviors, other studies are recommended with an emphasis on this dimension.

In the context of the relationship between the patients and the care provider, the results indicated that the training given was effective in this dimension. On the other hand, there was no significant change in the aspect of communicating about the symptoms of the disease. The results of the study by Rahmati et al., which have been conducted on other groups, have shown that training life skills is effective in balancing communication disorders, behavioral disorders and strengthening social adjustment and improving the social compatibility of children [19]. Regarding the content of the training and the results of previous studies, it was expected to see improvement in both dimensions; however, the results were not significant in terms of communication about the symptoms, which may be due to inadequate training in this dimension or the presence of other factors beyond the control of the researcher.

Generally speaking, the results of the research indicate that training life skills has a significant effect on the self-management of MS patients and self-management has significantly increased after education. So far, few studies have been conducted on behavior change to guide progress and improve interventions, and since this study has been conducted to improve knowledge and self-management behavior, it can help increase knowledge in this domain.

## Conclusion

Studies show that lack of awareness and knowledge about the self-management of disease and the related issues leads to various problems and different complications and reduces the quality of life. Considering the chronic form of the disease and the problems these patients face, drug therapy inefficiency in solving these difficulties and the need for continuous care, training can provide active and informed involvement of the patient in solving some of these problems. Thus, the main achievement of this study shows a positive effect of life skills training, because the primary goal is to prepare and help patients to solve problems resulting from the disease. This is not possible unless a reliable nurse-patient relationship is established to provide support, training and consultation. These pieces of training provide valuable information useful in nursing research as well as assessment and evaluation of care for MS patients.

## Acknowledgments

This research is registered by the Center of Clinical Trials with the code IRCT2016022326726 n1; it was also carried out with the support of the Iran University of Medical Sciences. The authors would like to thank the authorities of the MS Society in Tehran and the patients who collaborated in the study.

## Conflict of Interest

The authors confirm that there are no conflicts of interest.

## References

[1] Schmitt MM, Goverover Y, DeLuca J, Chiaravalloti N (2014). Self-Efficacy as a Predictor of Self-Reported Physical, Cognitive and Social Functioning in Multiple Sclerosis. *Rehabil Psychol*.

[2] Mcguire KB, Stojanovic-Radic J, Strober L, Chiaravalloti ND, Deluca J (2015). Development and effectiveness of a psychoeducational wellness program for people with multiple sclerosis: description and outcomes. *Int J MS Care*.

[3] Pagnini F, Bosma CM, Phillips D, Langer E (2014). Symptom changes in multiple sclerosis following psychological interventions: a systematic review. *BMC Neurol*.

[4] Maslakpak HM, Raiesi Z (2014). Effect of a Self-Management and Follow-Up Program on Self-Efficacy in Patients With Multiple Sclerosis: A Randomized Clinical Trial. *Nurs Midwifery Stud*.

[5] Peeters JM, Wiegers TA, Friele RD (2013). How technology in care at home affects patient self-care and self-management: a scoping review. *Int J Environ Res Public Health*.

[6] Aljumah M, Alroughani R, Alsharoqi I, Bohlega SA, Dahdaleh M, Deleu D (2013). Future of management of multiple sclerosis in the Middle East: A consensus view from specialists in ten countries. *Mult Scler Int*.

[7] Ploughman M, Austin MW, Murdoch M, Kearney A, Godwin M, Stefanelli M (2012). The Path to Self-Management: A Qualitative Study Involving Older People with Multiple Sclerosis. *Physiother Can*.

[8] Wollin JA, Fulcher G, Mcdonald E, Spencer N, Mortlock MY, Bourne M (2010). Psychosocial Factors That Influence Quality of Life and Potential for Self-Management in Multiple Sclerosis. *Int J MS Care*.

[9] Tan H, Yu J, Tabby D, Devries A, Singer J (2010). Clinical and economic impact of a specialty care management program among patients with multiple sclerosis, a cohort study. *Multiple Sclerosis*.

[10] Weinshenker BG (1994). Natural history of multiple sclerosis. *Ann. Neurol*.

[11] Plow MA, Finlayson M (2011). A Scoping Review of Self-Management Interventions for Adults with Multiple Sclerosis. *Self-Management Interventions for Adults with MS*.

[12] Vernooij RW, Willson MM (2016). Characterizing patient-oriented tools that could be packaged with guidelines to promote self-management and guideline adoption: a meta-review. *Implementation Science*.

[13] Thomas PW, Thomas S, Hillier C, Galvin K, Baker R (2009). Psychological interventions for multiple sclerosis. *The Cochrane Library*.

[14] Aparna N, Raakhee AS (2011). life skill education for adolescents: its relevance and importance. *Education Science and Psychology*.

[15] Bishop M, Frain M (2007). Development and Initial Analysis of Multiple Sclerosis Self-Management Scale. *Int J MS Care*.

[16] Rahmati B, Adibrad N, Tahmasian K, Sedghpour BS (2010). The Effectiveness of life skill training on Social adjustment in Children. *Procedia – Social and Behavioral Sciences*.

[17] Hailu TH, Shojaeizadehl D, Tol1 A, Garmaroudi Gh, Yekaninejad MS, Kebede A (2016). Psychological and Educational Intervention To Improve Tuberculosis Treatment Adherence in Ethiopia Based on Health Belief Model: A Cluster Randomized Control Trial. *PLOS ONE*.

[18] Jongen PJ, Heerings M, Ruimschotel R, Hussaarts A, Evers S, Duyverman L (2016). An intensive social cognitive program (can do treatment) in people with relapsing remitting multiple sclerosis and low disability: a randomized controlled trial protocol. *BMC Neurology*.

[19] Calandri E, Graziano F, Borghi M, Bonino SH (2016). Improving the quality of life and psychological well-being of recently diagnosed multiple sclerosis patients: preliminary evaluation of a group-based cognitive behavioral intervention. *Disabil Rehabil*.

